# Evaluation of Bond Strength of Resin and Non-resin Cements to Different Alloys

**DOI:** 10.7759/cureus.36894

**Published:** 2023-03-30

**Authors:** Navneet K Mann, Gagandeep K Chahal, Jaspinder Singh Gil, Sahil Kainth, Mitasha Sachdeva, Shagufta Verma

**Affiliations:** 1 Prosthodontics, National Dental College, Mohali, IND

**Keywords:** resin, alloy, bond strength, fixed prosthesis, metal-cement bond

## Abstract

Background: A strong metal-cement bond is one of the many factors that contribute to the clinical success of a fixed prosthesis. Even though it is crucial to create ideal resistance and retention forms during tooth preparation, dental cement must be strong enough to hold the restoration in place in the mouth. The present study set out to evaluate and compare the binding strength of resin modified glass ionomer cement, resin cement, and glass ionomer cement to four different metal alloys: titanium alloy, cobalt-chromium (Co-Cr) alloy, nickel-chromium (Ni-Cr) alloy, and noble metal alloys (silver palladium based).

Methods: Two hundred and forty metal alloy specimens were created; these were fashioned from (i) a noble metal alloy (silver-palladium based), (ii) a titanium alloy, (iii) a cobalt-chromium alloy, and (iv) a nickel-chromium alloy. A universal testing machine was used to perform the shear test, and statistical analysis of the result was done using a two-way analysis of variance (ANOVA) test and Bonferroni test.

Results:* *The Co-Cr alloy among the cement under investigation had the highest mean value of shear bond strength of 8.06 MPa, whereas a noble metal alloy had the lowest shear strength with a mean value of 5.36 MPa. The resin cement demonstrated the highest shear strength with a mean value that was higher than the other two types of cement. The shear bond strength of the examined samples was significantly affected by the interaction of the alloy and cement, according to the two-way ANOVA test (p=0.001).

Conclusion: The results demonstrate that resin cement offers a stronger bond, followed by resin-modified GIC and GIC. The Co-Cr alloy had the highest shear bond strength, followed by Ni-Cr, titanium, and noble metal alloy which showed significantly lower shear strength than the other three alloys.

## Introduction

To function properly, a permanent prosthesis needs a strong cement-metal bond. A luting agent's capacity to create a solid adhesive bond with a metal framework may help to lessen the effects of changes to the oral environment. One of the main factors contributing to the failure of fixed partial dentures is the bond's lack of strength [1-2]. Nickel, cobalt, and chromium alloys were created as base metal alloys. Ni-Cr alloys are the most often utilized alternative alloys for making crowns. These alloys are inexpensive and have good stiffness and hardness. Ni-Cr alloys are now being replaced with Co-Cr alloys, which seem to be more biocompatible. Titanium has become more widely used in cast restorations and fixed partial dentures, in large part due to advancements in casting tools and investment materials. Due to their superior marginal adaptability and ease of occlusal correction compared to base metal alloys, noble metal alloys are also becoming more and more popular [3].

For establishing long-lasting repairs, a wide variety of cementing chemicals are available. Due to its chemical adhesion to dentin and enamel, the release of fluoride, and thermal expansion coefficient that is comparable to the tooth structure, normal glass ionomer cement, for example, may have cariostatic potential [3-4]. Due to its significance for retention and long-term stability, adhesion has a significant impact on the clinical result of final restorations. The early stages of the setting process might result in moisture damage to this material because of its weak tensile strength, ease of breaking, and susceptibility to breaking [3]. Manufacturers started marketing "resin-modified glass ionomer cement" in the late 1980s. This product combined the benefits of glass ionomer cement with a resin composite [4]. Comparing this material to zinc phosphate and other common glass ionomers, it exhibits stronger compressive and diametral tensile strengths. Because resin-modified glass ionomer cement is hydrophilic, it absorbs more moisture and expands in wet environments [5]. Either photochemically or chemo-chemically may be used to polymerize dual-polymerizing resin cement. These luting materials' two polymerizing processes allow for the final cementation of all indirect ceramic, composite, and metal restorations. Additionally, dual-polymerizing resin cement provides outstanding mechanical strength and wonderful esthetic attributes [5]. A poor metal-cement bond may cause the cement at the finish line margin to dissolve, which causes micro-leakage, secondary caries, crown dislodging, periodontal issues, and even devitalization of the abutment [6]. The kind of luting cements utilized, its thickness, the nature of the planning, the idea behind the amalgam, how the surfaces are handled, and the pressures the prosthesis must withstand all play a part in the patient's discontent [7]. In order to prevent dislodgement caused by interfacial or cohesive failures, dental cement must be able to form a long-lasting bond between incompatible materials and have good compressive and tensile strengths, resistance to disintegration in the mouth cavity, and adequate fracture toughness [8].

The discrepancies between the main constituents may have an impact on the luting cement's and the tooth substance's ability to bond. The clinical success of dental restorations depends heavily on the luting cement and the cementation techniques. The main goals of a dental luting agent are to make the restoration's bond to the tooth stronger and to prevent it from fracturing. Selecting the proper luting agent is essential since a strong binding to the key components is so crucial [9]. This study's objective was to assess the shear bond strength of four different types of noble metal alloys (silver palladium-based, titanium, cobalt chrome, and nickel chrome) in comparison to resin cements, glass ionomer cements, and resin-modified glass ionomer cements.

## Materials and methods

**Figure 1:**
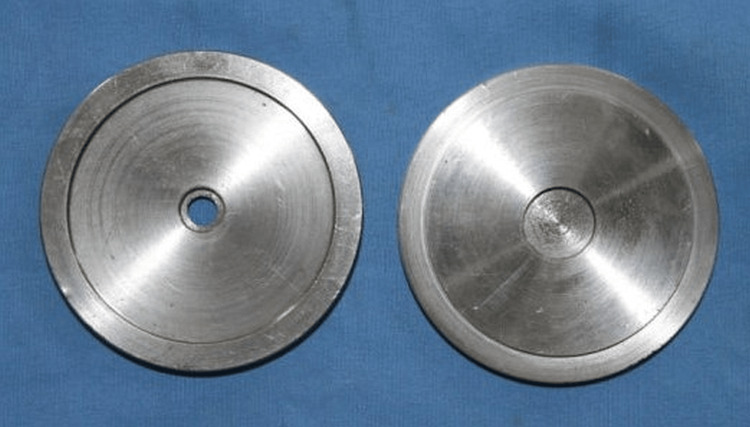
Representation of the metal die.

For the present study, the sample size was calculated using the G power software (Heinrich Heine University, Düsseldorf, Germany), as at least 20 specimens per group for a significance level of α = 0.05 and power of 0.82 was confirmed. A metal dies, 33 mm x 36 mm, consisting of a base, middle, and top part, was fabricated. The middle and top parts were 4 mm thick and had a 10 mm hole in the center. Metal discs with a thickness of 4 mm and a diameter of 10 mm were made by using the base and the central section of the die (Figure 1).

**Figure 2:**
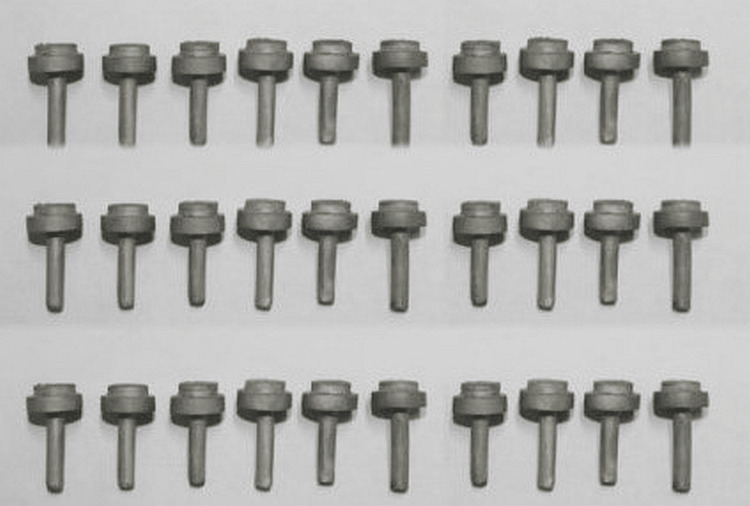
Casted samples used.

All samples were thermocycled after being incubated in distilled water at 37 degrees Celsius for 24 h. We stored the samples for six months at 37 degrees Celsius in distilled water. Some 2000 heat cycles were performed on the finished items in water baths (5°C-55°C) with dwell times of 30 s and transfer times of 10 s. The shear bond strength was determined with the use of a universal testing machine (INSTRON LR-100, Bangalore, India) (Figure 2).

**Figure 3:**
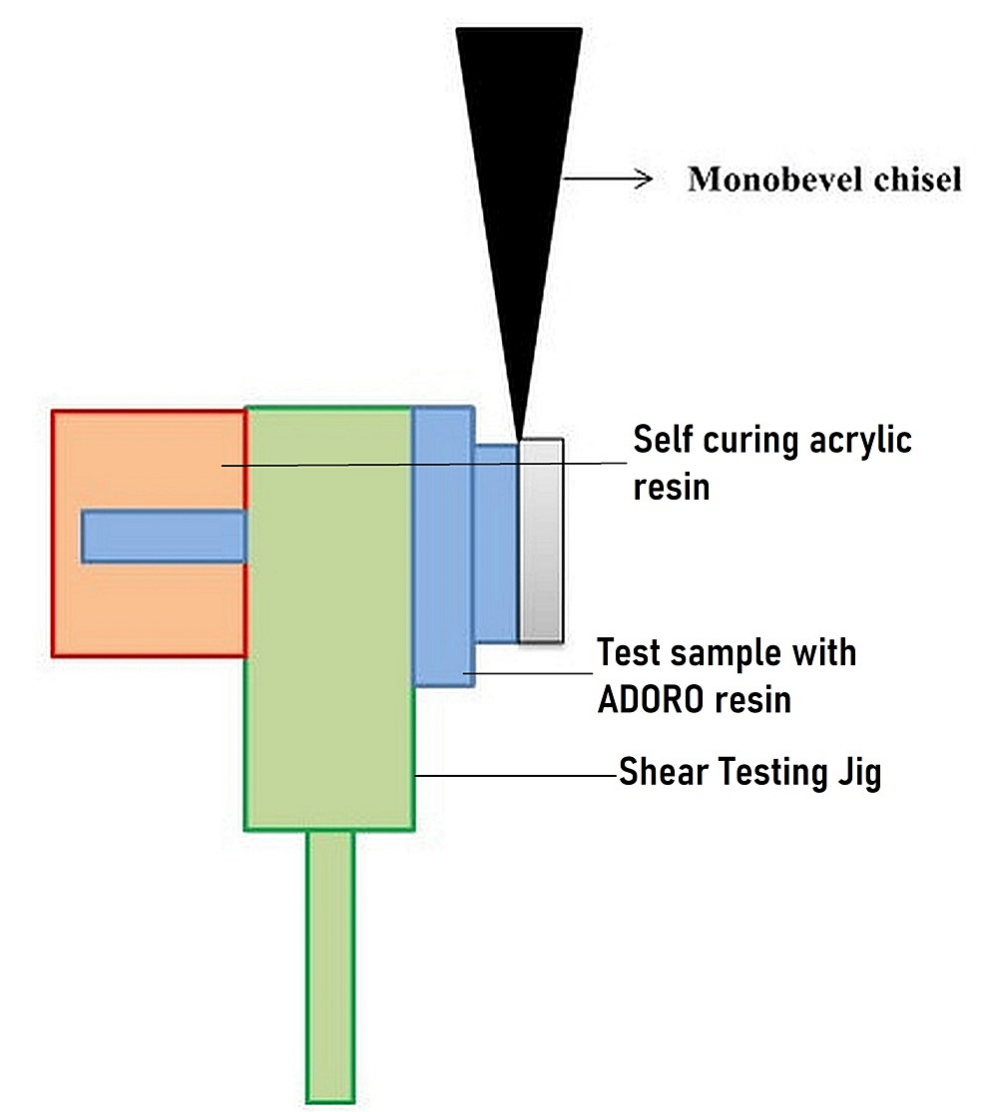
Schematic representation of the testing of the bond strength.

Over the treated surface of the cast pattern, universal wax (Dentic All wax No. 702-0500, Renfert, Germany) was added to a thickness of 2 mm. In order to make a mold space, the metal sample and wax-up were embedded in additional polymerization polyvinyl siloxane impression material of putty consistency (Reprosil Vinyl Polysiloxane Impression Material) (Dentsply, CA, USA). The stone dies were produced after Type IV dental stone (GC Asia Dental, Medak District, Telangana, India) was poured into the mould cavity. The matrix created in this way had space for the SR ADORO resin cement, which was later added over the metal casting as directed by the manufacturer. The shear bond strength of the completed samples was assessed using the Instron Universal testing apparatus after they had been mounted in a jig. A total of 240 machined rectangular jigs with a handle on one end and a through hole on the other end were manufactured. The jigs had dimensions of 10 mm by 4 mm. Because of the hole in one of the ends, the metal-resin test sample could be inserted from one side, and it was held in place with the assistance of self-curing acrylic resin on the other side (DPI-RR Cold Cure, Dental Products of India, Mumbai, India). The samples were mounted in the universal testing machine with the help of the handle located on the opposite end. In the shear testing jig, the test samples are attached in the appropriate positions. On a Universal testing machine, the mounted samples were examined to determine the shear bond strength. At the point where the metal and the resin meet, a shearing force was applied to the test sample using a mono bevel chisel that had been custom-made. After loading the specimens, shear bond strength was determined by moving the crosshead at a speed of 1 mm/min. The load was recorded on a computer that was connected to the testing equipment. Megapascals were the units of measurement that were used. To avoid the bias in the study, one post-graduate researcher carried out the procedure (Figure 3).

Two hundred and forty metal alloy specimens were created, each measuring 10 mm in diameter and 4 mm in thickness. These were fashioned from Group I: noble metal alloy (silver-palladium based, Elektra, Ivoclar, United States of America), Group II: titanium alloy (Titanium Inc., Polgate, England, UK), Group III: cobalt-chromium alloy (Diadur, DFS, Germany), and Group IV: nickel-chromium alloy (Bellabond, Bego, Germany). The samples were separated into four categories based on the metals they were made from and subdivided into Subgroup Ia: The specimens were coated with resin cement with metal primer (Panavia F, Kuraray, Japan). Subgroup Ib: GC FujiCEM (GC Corporation, Bunkyo-ku, Tokyo, Japan) was used on specimen surfaces. Subgroup I c: GC Gold Label glass ionomer cement covers the specimens (GC Corporation, Bunkyo-ku, Tokyo, Japan).

The data (MPa) were analyzed using a two-way ANOVA and Bonferroni test at a significance level of 0.05 to study the significant of various in means among groups using Statistical Package for the Social Sciences (SPSS) version 18 (IBM Co., Armonk, NY, USA).

## Results

The descriptive statistics of shear bond strength according to alloys and cement materials as shown in the Table 1 and represented in Figure 1.

**Table 1:** Descriptive statistics of shear bond strength according to alloys and cement materials. SD, standard deviation; GIC, glass ionomer cement; RMGIC, resin modified glass ionomer cement; Co-Cr, cobalt-chromium alloy; Ni-Cr, nickel-chromium alloy

Alloy	Cement	Mean strength	SD
Noble metal	GIC	2.72	0.18
	RMGIC	3.47	0.25
	Resin cement	5.36	0.25
Titanium	GIC	3.46	0.27
	RMGIC	4.20	0.32
	Resin cement	5.61	0.33
Co-Cr	GIC	5.11	0.84
	RMGIC	6.76	0.64
	Resin cement	8.06	0.57
Ni-Cr	GIC	4.98	0.54
	RMGIC	6.24	0.54
	Resin cement	7.69	0.40

**Figure 4:**
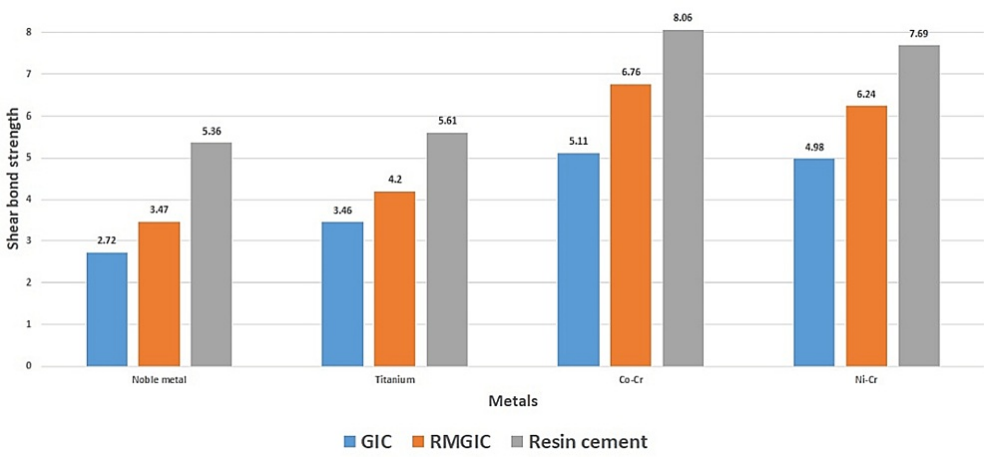
Bar diagram to show the comparison of shear bond strength.

Different alloy materials showed a significant effect on shear bond strength. Difference cements showed a significant effect on shear bond strength. Two-way ANOVA test showed a significant effect of interaction of alloy and cement on shear bond strength of tested samples (Table 2).

**Table 2:** Evaluation of effect of interaction of cement and alloy on shear bond strength. Two-way ANOVA test *indicates significant difference at p ≤ 0.05; ^a^R Squared = 0.926 (adjusted R squared = 0.922) ANOVA, analysis of variance; SD, standard deviation

Tests of between-subjects effects					
Dependent variable: shear bond strength					
Source	Type III sum of squares	Df	Mean square	F	Sig.
Corrected model	622.873^a^	11	56.625	259.216	0.001*
Intercept	6752.416	1	6752.416	30911.065	0.001*
Alloy	340.345	3	113.448	519.342	0.001*
Cement	275.168	2	137.584	629.829	0.001*
Alloy * cement	7.360	6	1.227	5.615	0.001*
Error	49.806	228	0.218		
Total	7425.095	240			
Corrected total	672.679	239			

Co-Cr alloy showed significantly higher shear strength than other three alloys. Ni-Cr alloys showed significantly higher shear strength than noble metal alloy and titanium alloy and titanium alloy showed significantly higher shear strength than noble metal alloy. Noble metal alloy showed significantly lower shear strength than other three alloys (Table 3).

**Table 3:** Pairwise comparison of shear bond strength among different alloys. Adjustment for multiple comparisons: Bonferroni test; *indicates significant difference at p ≤ 0.05 Ti, titanium alloy; Co-Cr, cobalt-chromium alloy; Ni-Cr, nickel-chromium alloy

(I) Alloy	(J) Alloy	Mean difference (I-J)	Standard error	p value	95% CI for difference	
					Lower	Upper
Co-Cr	Ni-Cr	0.342	0.085	0.001*	0.115	0.569
	Noble metal	2.792	0.085	0.001*	2.565	3.019
	Ti	2.219	0.085	0.001*	1.992	2.446
Ni-Cr	Noble metal	2.450	0.085	0.001*	2.223	2.677
	Ti	1.877	0.085	0.001*	1.650	2.104
Noble metal	Ti	-0.573	0.085	0.001*	-0.800	-0.346

GIC showed significantly lower shear strength than other two cements and resin cements showed significantly higher shear strength than other two cements (Table 4).

**Table 4:** Pairwise comparison of shear bond strength among different cements. Adjustment for multiple comparisons: Bonferroni test; *indicates significant difference at p ≤ 0.05 GIC, glass ionomer cement; RMGIC, resin modified glass ionomer cement

(I) Cement	(J) Cement	Mean difference (I-J)	Standard error	p value	95% CI for difference	
					Lower	Upper
GIC	Resin	-2.612	0.074	0.001*	-2.790	-2.434
	RMGIC	-1.101	0.074	0.001*	-1.279	-0.923
Resin	RMGIC	1.511	0.074	0.001*	1.333	1.689

## Discussion

In a fixed restoration, the cohesiveness of the luting cement, adhesion between the intermediate luting cement and the tooth surface, and adhesion between the cast alloy and the luting cement are the three factors that determine bond strength [10]. Acrylic or diacrylate resin serves as the foundation for adhesive resin cement, which also includes adhesive monomers that adhere effectively to metal substrates. If using adhesive resin cement to attach to ceramic, metal, or dental surfaces, a separate primer may be necessary. Despite the fact that adequate bonding has been achieved in the clinic at the latter two locations, the adhesion between the cast alloy and the cement is rather variable and a frequent site of failure [11]. The effectiveness of dental cement's adhesion to different dental metals was thus tested by researchers.

Any of the luting cement that is commercially available may be used to bond the tooth structure and the restorations together. Despite having several significant disadvantages, the most popular type of cement is zinc-phosphate cement. High solvency, weak adherence, and the absence of a compound bond to the substrate are a few of these. In our study, the resin cement exhibited the highest shear strength of the other two types of cement, of which the Co-Cr alloy had the shear bond strength with the highest mean value of 8.06 MPa, whereas a noble metal combination had the lowest value of 5.36 MPa. When the two-way ANOVA test was performed to test the null hypothesis among the alloy groups, a statistical difference of p=0.001 was seen. This notion is supported by the idea that oxides might develop on central metals at room temperature and that these oxides are more sensitive than cement. The oxides on the metal's surface might potentially be used as compound storage sites in addition to giving the metal a rougher appearance. It is more difficult to form a thin oxide deposit at the surface of a noble compound, so it has lower bond values. The improved bond quality in these mixtures may be due to the base metals' greater free-surface energy and oxide-framing capability, despite the fact that they are more sensitive than noble compounds [10]. It has been shown that bond strength decreases as the proportion of noble metals in alloys rise because less oxygen is present in the surface oxide layer. This emphasizes how important the oxides produced on the metal surface are for promoting adhesion. Compared to the Ni-Cr alloy, the Co-Cr alloy demonstrated a stronger bond. The higher chromium concentration of the Co-Cr alloy provides evidence in favor of this [10]. According to research by Salonga et al. [12], this chromium produces chromium oxide, which creates bonds that are more powerful than those created by other metal oxides.

Sen et al. [13] discovered that bond strength rose with increasing base alloy components, which is consistent with the theory that base metal alloys have a better potential to produce oxides. There is concordance between this study and recent studies. According to studies by Capa et al. [9], resin cement or resin-modified glass ionomer cement has been proven to have a better bond strength with base metal alloys than with noble metals. Our recent study produced comparable outcomes. According to Ohkubo et al. [14], the Co-Cr alloy has a poorer binding strength to self-curing resin when compared to titanium alloy. The findings of this research are in concordance with those of the current study. This could be a result of the specimens utilized in their investigation simply having undergone grit blasting, with no further oxidation. Grit-blasted titanium alloys exhibit rougher and more abrasive surfaces than Co-Cr alloys [10]. However, chromium oxide, which has a larger bonding potential, is created when the metal is oxidized once again, as was done in this work.

Compared to other types of cement, resin cement has a greater average shear bond strength. This theory is supported by the phosphoric acid methacrylates present in resin cement, which provide a strong physical interaction with the metals (comparable to hydrogen bonding) [10]. With the aid of adhesive monomer, resin cement creates a solid, long-lasting bond. Perhaps this is as a consequence of the resin-based agents' post-polymerization, which produced even stronger linkages. The glass ionomer cement has the lowest mean shear bond strength. This is due to the fact that it is quickly decomposed by the water, which reduces its hardness and makes it simpler to dissolve. In a related investigation, Piwowarczyk et al. found that resin cement exhibited high binding strengths to prosthodontic materials [5]. When compared to glass ionomer cement, resin-modified glass ionomer both showed lower values. The results of this investigation supported those of Capa et al., who found that resin cement offered improved bonding to a variety of metal alloys in comparison to resin-modified glass ionomer cement [9]. Therefore, resin cement may be used to combine different core materials together. According to studies by Mojon et al., resin cement has the strongest binding with tooth metals, but glass ionomer cement has a lesser value [15]. 

Reza and Lim used a push force to assess the durability of self-cured and dual-cured resin cement on a titanium post [16]. They discovered that glass ionomer luting cement that was resin-modified and self-cured had a stronger bond than resin cement. The findings of this research did not match with those of the current one. The use of titanium endodontic posts in their study is one theory. Studies using resin-luting cement, which has been utilized to close the gaps between the tooth's enamel and the tooth's hard, enamel-like root canal lining, have found root canal wall voids and bubbles. These gaps and bubbles unquestionably weaken the bonding strength when endodontic posts are joined with resin cement. The greatest average bond strength was seen in the Co-Cr alloy among metals, and in resin cement among cements [17-23]. The limitations of the study include the production of the metal pellets, the casting methods, and the cementation processes all varied. To further understand these events, more research is necessary. Studies on the effects of fatigue and cyclic loads are also necessary in order to comprehend the long-term durability of this method.

## Conclusions

This research compared resin cement, resin-modified GIC, and GIC in shear bond strength to various metals (noble metal alloy, titanium alloy, Co-Cr alloy, and Ni-Cr alloy). In this study, the Co-Cr alloy which had the highest shear bond strength, followed by Ni-Cr, titanium, and noble metal alloy showed significantly lower shear strength than the other three alloys. Resin cement exhibited the highest shear strength, followed by resin-modified GIC and GIC. Based on these findings, a fixed prosthesis success may be ensured by carefully selecting the luting agent and alloy material.
